# Supplementary material for: Genome-based metabolic and phylogenomic analysis of three Terrisporobacter species
Source: PLoS One. 2023 Oct 10;18(10):e0290128. doi: 10.1371/journal.pone.0290128 (PMC10564238; doi:10.1371/journal.pone.0290128)
Supplement: S1 Table — (DOCX) [file pone.0290128.s001.docx]

**S1 Table**. **Kits and program versions for Nanopore sequencing.**

|  | *T. mayombei* DSM 6359^T^ | *T. petrolearius* JCM 19845^T^ | *T. glycolicus* DSM 1288^T^ |
| --- | --- | --- | --- |
| Ligation sequencing kit 1D | SQK-LSK108 | SQK-LSK109 | SQK-LSK109 |
| Barcoding kit | EXP-NBD103 | - | EXP-NBD114 |
| MinKNOW | v2.2 | v21.10.4 | v22.10.7 |
| Basecalling software | Albacore in MinKNOW v2.2 | Guppy v6.0.1 (HAC mode) | Guppy v6.4.2 (HAC mode) |
