# Supplementary material for: Genome-based metabolic and phylogenomic analysis of three Terrisporobacter species
Source: PLoS One. 2023 Oct 10;18(10):e0290128. doi: 10.1371/journal.pone.0290128 (PMC10564238; doi:10.1371/journal.pone.0290128)
Supplement: S2 Table — ANIm percentage identity values (white, in %) and corresponding alignment lengths (grey, in bp) for available Terrisporobacter genomes at NCBI Genbank. (DOCX) [file pone.0290128.s002.docx]

**S2 Table**. **ANIm percentage identity and alignment length values.** ANIm percentage identity values (white, in %) and corresponding alignment lengths (grey, in bp) for available *Terrisporobacter* genomes at NCBI Genbank.

|  | **KPPR9** | **HGM13572** | **TEPE** | **08-306576** | **TEMA** | **UN03-225** | **DSM 29186** | **ban5-GNNATD2-55** | **MSK41** | **TEGL** | **MCA3** | **FS03** | **bin132** | **WW3900** | **MAG180** | **MGYG-HGUT-00005** | **bin55** | **bin150** | **bin76** | **bin336** | **UBA8115** |
| --- | --- | --- | --- | --- | --- | --- | --- | --- | --- | --- | --- | --- | --- | --- | --- | --- | --- | --- | --- | --- | --- |
| **KPPR9** | 3985435.0 | 87.24 | 96.35 | 87.79 | 92.21 | 98.85 | 87.93 | 85.16 | 87.78 | 93.03 | 98.91 | 98.30 | 85.60 | 98.46 | 85.13 | 98.78 | 85.38 | 85.38 | 85.51 | 85.60 | 97.80 |
| **HGM13572** | 351735.0 | 1643249.0 | 87.10 | 87.62 | 87.07 | 87.22 | 87.30 | 86.47 | 87.34 | 87.00 | 87.23 | 87.25 | 99.17 | 87.24 | 86.54 | 87.32 | 99.16 | 88.45 | 99.16 | 99.14 | 87.14 |
| **TEPE** | 3324202.0 | 355860.0 | 4124338.0 | 87.91 | 92.34 | 96.43 | 88.01 | 85.06 | 87.86 | 93.09 | 96.35 | 96.31 | 85.62 | 96.31 | 85.15 | 96.33 | 85.37 | 85.38 | 85.51 | 85.60 | 95.82 |
| **08-306576** | 2424641.0 | 408743.0 | 2418115.0 | 3986874.0 | 88.37 | 87.92 | 99.62 | 85.35 | 99.74 | 88.18 | 87.90 | 87.90 | 86.33 | 87.87 | 85.57 | 87.85 | 86.12 | 85.74 | 86.21 | 86.16 | 87.70 |
| **bin39** | 1176493.0 | 624408.0 | 1167052.0 | 1258161.0 | 85.36 | 85.36 | 85.91 | 85.00 | 85.73 | 85.36 | 85.31 | 85.33 | 99.00 | 85.29 | 84.82 | 85.30 | 99.05 | 86.69 | 99.44 | 99.01 | 85.32 |
| **TEMA** | 3156839.0 | 366658.0 | 3154364.0 | 2550285.0 | 4064271.0 | 92.30 | 88.50 | 85.28 | 88.28 | 93.48 | 92.33 | 92.25 | 85.61 | 92.24 | 85.17 | 92.26 | 85.40 | 85.47 | 85.53 | 85.59 | 91.77 |
| **UN03-225** | 3515635.0 | 351593.0 | 3265070.0 | 2384107.0 | 3124676.0 | 3806082.0 | 88.01 | 85.20 | 87.88 | 93.06 | 98.67 | 98.27 | 85.65 | 98.29 | 85.21 | 98.58 | 85.46 | 85.50 | 85.59 | 85.64 | 97.77 |
| **DSM 29186** | 2332450.0 | 389632.0 | 2329192.0 | 3623251.0 | 2464896.0 | 2292871.0 | 4058004.0 | 85.52 | 99.72 | 88.31 | 88.00 | 88.00 | 86.16 | 87.96 | 85.60 | 87.99 | 85.98 | 85.88 | 86.11 | 86.12 | 87.81 |
| **ban5-GNNATD2-55** | 648111.0 | 173397.0 | 647132.0 | 705438.0 | 659879.0 | 637328.0 | 679806.0 | 1385053.0 | 85.27 | 85.27 | 85.13 | 85.11 | 85.13 | 85.16 | 92.00 | 85.17 | 85.18 | 85.03 | 85.10 | 85.15 | 85.04 |
| **MSK41** | 2435937.0 | 399658.0 | 2437470.0 | 3755138.0 | 2554078.0 | 2401191.0 | 3642481.0 | 705008.0 | 4061113.0 | 88.11 | 87.85 | 87.86 | 86.03 | 87.84 | 85.50 | 87.85 | 85.81 | 85.72 | 85.94 | 85.98 | 87.68 |
| **TEGL** | 3187079.0 | 377106.0 | 3181191.0 | 2596099.0 | 3310666.0 | 3145846.0 | 2494419.0 | 648933.0 | 2618600.0 | 4060596.0 | 93.09 | 93.02 | 85.68 | 93.00 | 85.20 | 93.00 | 85.41 | 85.44 | 85.57 | 85.61 | 92.54 |
| **MCA3** | 3560710.0 | 355929.0 | 3350632.0 | 2435982.0 | 3201225.0 | 3520457.0 | 2362870.0 | 653707.0 | 2451248.0 | 3275077.0 | 4116837.0 | 98.32 | 85.60 | 98.55 | 85.12 | 99.02 | 85.41 | 85.36 | 85.52 | 85.58 | 97.81 |
| **FS03** | 3549400.0 | 357286.0 | 3332103.0 | 2425955.0 | 3155528.0 | 3529491.0 | 2334797.0 | 652666.0 | 2446025.0 | 3225080.0 | 3609201.0 | 3947610.0 | 85.59 | 98.21 | 85.20 | 98.34 | 85.42 | 85.28 | 85.51 | 85.59 | 98.91 |
| **bin132** | 1277204.0 | 641150.0 | 1275428.0 | 1370704.0 | 1313621.0 | 1260626.0 | 1297729.0 | 604067.0 | 1349708.0 | 1284990.0 | 1282522.0 | 1283546.0 | 2667940.0 | 85.57 | 85.01 | 85.56 | 99.26 | 86.65 | 99.16 | 99.81 | 85.48 |
| **WW3900** | 3505226.0 | 352759.0 | 3288328.0 | 2419998.0 | 3102971.0 | 3480051.0 | 2327282.0 | 637552.0 | 2429905.0 | 3176340.0 | 3556564.0 | 3563538.0 | 1283334.0 | 3880773.0 | 85.16 | 98.56 | 85.41 | 85.36 | 85.53 | 85.56 | 97.81 |
| **MAG180** | 985288.0 | 277630.0 | 1005726.0 | 1180726.0 | 1068361.0 | 979779.0 | 1158821.0 | 1116575.0 | 1197821.0 | 1089204.0 | 1013623.0 | 998913.0 | 924232.0 | 988201.0 | 3255212.0 | 85.20 | 84.87 | 84.72 | 84.97 | 85.015 | 85.24 |
| **MGYG-HGUT-00005** | 3558020.0 | 350096.0 | 3331010.0 | 2432724.0 | 3147471.0 | 3512186.0 | 2338077.0 | 645189.0 | 2445508.0 | 3224646.0 | 3677367.0 | 3592166.0 | 1291533.0 | 3575105.0 | 1000880.0 | 3932530.0 | 85.42 | 85.34 | 85.52 | 85.58 | 97.86 |
| **bin55** | 1249687.0 | 684589.0 | 1235486.0 | 1340111.0 | 1281794.0 | 1230170.0 | 1286284.0 | 606158.0 | 1334780.0 | 1290867.0 | 1249457.0 | 1245028.0 | 2471338.0 | 1241757.0 | 905403.0 | 1247657.0 | 2906149.0 | 86.95 | 99.23 | 99.25 | 85.39 |
| **bin150** | 1060863.0 | 338104.0 | 1061910.0 | 1178170.0 | 1096119.0 | 1033656.0 | 1135889.0 | 496603.0 | 1192057.0 | 1083060.0 | 1066966.0 | 1070572.0 | 1221322.0 | 1069440.0 | 712440.0 | 1067437.0 | 1253047.0 | 2219718.0 | 86.74 | 86.68 | 85.34 |
| **bin76** | 1264290.0 | 677912.0 | 1251031.0 | 1350328.0 | 1284333.0 | 1245628.0 | 1289240.0 | 602588.0 | 1334798.0 | 1282316.0 | 1262923.0 | 1264140.0 | 2466965.0 | 1267559.0 | 908537.0 | 1269894.0 | 2583546.0 | 1227605.0 | 2748600.0 | 99.15 | 85.44 |
| **bin336** | 1279801.0 | 653866.0 | 1275912.0 | 1353858.0 | 1318947.0 | 1266292.0 | 1303054.0 | 602706.0 | 1351374.0 | 1307172.0 | 1286239.0 | 1284823.0 | 2512868.0 | 1289758.0 | 922790.0 | 1289048.0 | 2534538.0 | 1231002.0 | 2540203.0 | 2728736.0 | 85.50 |
| **UBA8115** | 3349193.0 | 313165.0 | 3105141.0 | 2170967.0 | 2947219.0 | 3294678.0 | 2127810.0 | 591789.0 | 2181204.0 | 3024118.0 | 3403299.0 | 3470811.0 | 1132108.0 | 3315076.0 | 885831.0 | 3371261.0 | 1117334.0 | 934780.0 | 1133504.0 | 1137658.0 | 3837521.0 |
